# Cora’s Delusion

**Published:** 1874-11

**Authors:** 


### CORA'S DELUSION.

Cora Depeyster pined for a prince. Not a common prince like the snuffy old Russian we used to see on Broadway, wearing a greasy fur-collar, and long overcoat, eating garlic and drinking brandy three times diurnally, and growling at everything American in the most detestable English. Nothing of the kind.

She pined for a prince such as we read about in the fine old sentimental novels that amused our fathers and mothers in the flower of their youth ; a Thaddeus of Warsaw, all talent, and pallor, and tenderness, and musical voice, and fine, rolling eyes, and pedigree, and that sort of thing.

For my part I don't believe in such princes. The Prince of Wales isn't one of that species, and I'm afraid the breed has gone out with the fine old sentimental novels. Nothing else, however, would suit Miss Cora Depeyster. Being not totally unlike other fair damsels of twenty or thereabouts, she desired to experiment upon the state matrimonial, and had plenty of opportunities, but common clay would not do. A prince she must have or single she would remain.

Now, a real good writer of sentimental stories could create such a being especially for the emergency, and, after the customary amount of tantalization through the medium of an obstinate parent or what not, marry off Miss Cora and her prince in the most satisfactory style. But I never was good at sentimental creation. I must write about people I know and see. I am sorry for the Coras. I've seen lots of them ; but what sort of princes did they marry ? One now pours tea for a sharp-nosed, red-haired life-insurance agent. Another is the spouse of a strapping farmer, who sits with hat on and eats in his shirt-sleeves. Another wedded a subdued German, who plays second clarionet in a cheap orchestra, and has to move monthly because he can't pay his rent. Still another—but the catalogue grows melancholy. Thus with all the Coras. They go on pining for princes that never come ; marrying all sorts of people instead ; and, dying, give way to a fresh race of Coras, who follow in their mamma's footsteps with a disregard of experience that savors of the sublime.

One of the sweetest of watering-places is Happy Valley. It is romantic and comfortable at once. There is delightful bathing, rowing, sailing, and fishing in the lake itself, and the shady groves that line its shores are cool and green and mysterious, and suggestive of dryads and nymphs and fairies and things. That is, if you happen to be of a poetic turn of mind. If not, they only suggest flirtations.

I will not further expatiate upon the delights of Happy Valley, lest it should be fancied that I have lots for sale in the vicinity, whereas I have none anywhere —not even a burial lot ; and that, I believe, is the common lot of all. Neither did Cora Depeyster have any real estate to dispose of ; but she thought just as I do, and passed much of the time every summer in the fair demesnes that lie smilingly adjacent to the Happy Valley Pavilion.

Notwithstanding the round of pleasures in which she every summer took prominent part, she could not stifle her inward and continuous yearning for the music of a princely voice, the glance of devotion from princely eyes, the touch of a princely hand in the dance, and the sweet envy of all maidens who had to go princeless through life. She sighed a great deal, and began to think the great world a cold, hard, unromantic sort of an arrangement.

Of course, you and I know better. I never wrote a romance yet half so wonderful as the simplest life would make were it truly told. The only trouble is that the simplest life cannot be truly told. It seems easy, but you try it once !

Though there was no prince among the Pavilion boarders, there was a poet. Arthur Bayne was there. It is barely possible that Cora might have fallen in love with him ; there is something very fine and hyfalutin—pardon the expression—in a young lady's idea of a poet. But then Arthur Bayne was altogether different from her ideal. He knew the world too well to believe in its hollowness. He had found it in fact a very round, hard, and stubborn sort of thing. I notice that men who have really been shaken up a bit in the merry-go-round we call life, are not apt to preserve the outside show of sentimentalism to any great extent. We all start off, some time or other, with our long hair, our turn-down collars, our stable suits, our brigand hats, and our little hidden sorrows ; but when we have cut our eye-teeth, and learned something about other folks' trouble, we always come back to reason, to plain neckties, to stove-pipe hats, and the barber.

Arthur was too much matter of fact for Cora, and she too sentimental for him from any hymeneal point of view, yet they somehow became very excellent and very intimate friends. One evening they sat on the shore of the little lake together. Cora was gazing at the moon, of course. She was one of that kind. She had been telling Arthur what kind of a hero she had imagined for her heart history, and described the prince with his melting eyes and musical voice, his generous nature and magnificent air, his mild melancholy and inexhaustible affection, his irreproachable morals and aristocratic birth. Arthur listened with due gravity until she had finished.

"Why don't you take me?" he asked. "I am not very rich, but then poverty is romantic. I can't play the guitar, but I know a fellow who is splendid on the banjo. As for a fine antique family, my father was Mr. Bayne, son of Old Bayne; I believe he never went to State prison."

"Now, Arthur, you are too bad! You make fun of everything."

"Well, to be serious, child, you'll never find your prince."

"And why not?"

"Because they don't make 'em. Most men are tolerably human, and humanity is not perfection. If a man has no other small, vices, he is sure to chew tobacco write poetry, or keep a dog. We are fair, but frail, we men."

"Ah, Mr. Bayne, what a world it is! I wish there might be some place where one might go and hide away and dream in peace."

"There is; and I am going there now. I refer to my bed."

The next morning the belles of Happy Valley were in a twitter. The late train the night before had brought a new young man, and young men were not a drug at the Pavilion. The new comer had taken the finest suite in the establishment, and a great pile of trunks with his initials stood in the vestibule, where they were jealously regarded by the other young men, heart-broken with the consciousness of possessing but a single trunk, and that perhaps a small one. When it became known that the unknown was really on the piazza, smoking a cigar, all sorts of diplomatic manœuvres were resorted to to get a fair sight of him on the part of the young ladies. Cora Depeyster denounced it as an exhibition of brazen ill-breeding in others. As for herself, she only stood at the window which commanded the piazza, and scrutinized him through an opera glass.

Montgomery Smythe—for such was the name which appeared on the register—in three days' time found himself a favorite with the ladies. He was of the conventional type of magnificence—the black-haired, black-eyed, red-cheeked style, with small feet, dyed moustache, and eye-glasses. In the matter of scarfs and neckties, with the jeweled pins thereto devoted, he was truly gorgeous. If a man has the least taint of vulgarity let him beware of his neck. Too much thoracic decoration ruins one.

A great change came over Cora. She was sad and gay by fits; irritable, changeable, and incomprehensible.—There is no use wasting words about it. She was in love. Her prince had come.

As the days wore on this regal person developed. He gave suppers in his room to the young bloods, and organized pic-nic parties in the woods thereabout, which made the belles of the Pavilion quite miserable with happiness. To Cora's intense delight he made her in some sort the central figure in these last charming affairs, and held profound consultations with her concerning the details. They thus became associated in a certain degree before the public eye, and when rumor whispered an engagement, Cora did little more than blush and stammer a denial that sounded ever so much like a confirmation.

She gave herself up to a sort of blind adoration of Montgomery Smythe. She made a prince of him first, and put all her trust in him afterward. He told her of his ancient family; of his late father, Judge Dewey, twice United States Senator, and son of Commodore Smythe, of the war of 1812. The Commodore's father, he said, was General Smythe, of revolutionary fame, and brother to Governor Smythe, of one of the colonies under George III. He talked of the magnificent old country-seat his father had left him, with its picture-gallery full of the portraits of the old worthies just mentioned, and their wives; all uniforms and brocades and gold braid and laces; of the long drawing-rooms, the grand dining-hall, the library, the grounds—all in true baronial style, till Cora, rich and luxuriously reared as she was, began to look up to him as a being of an altogether different and higher sphere.

One day they took a walk in the grove in the rear of the Pavilion. It was the closing up of the season, and the next day there was to be a general exodus of the Happy Valley boarders, to their homes. Cora felt that the decisive moment had arrived; and it had. The hitherto pent-up devotion of Montgomery Smythe found vent at last in a declaration and a proposition. He vowed his love in a perfectly princely style, and having been accepted with a good many blushes and tears, just as is the case in all well-written novels, he informed her that letters just received from his confidential agent in Europe compelled him to start immediately for Paris, and urged her to marry him at once, without waiting to go through the form of asking the permission of her grandsire or consulting her friends. Was he not Montgomery Smythe ? and who could possibly object to such an alliance ?

It is very possible that Cora might have consented, so infatuated was she with her prince ; but she had read that the regular thing was to demand time for consideration, so she postponed her decision, which really was already made, until evening.

As they reached the piazza, he lazily tapping his glossy boot with his bamboo, and she very tremulous and very happy, a thick-set, pock-marked individual, with black, heavy whiskers and a glazed cap, came down the steps, and, nodding to Smythe, said :

"I'd like to say a private word to you, young man."

Montgomery Smythe suddenly stopped tapping his boot and, turning pale, looked sharply at the stranger. A slight vibration of that person's eyelid made him turn still paler, and without a word he walked several steps away from the Pavilion. The stout man then slowly drew a large pocket-book from his breast, favored Smythe with a view of certain documents therein contained, immediately after which he said aloud :

"You're my prisoner, sir, in the name of the law !"

Cora felt like fainting, but her curiosity was more than a match for her weakness.

Smythe looked toward her, laughed a little, gasping laugh, and tried to say that this ridiculous mistake could be easily explained.

"Let this person explain it, then," said Cora, trembling all over.

"Why, you see, miss," said the stout man, "I'm a detective officer, and I've been laying for this young gentleman some time. I have his photograph here, miss, if you'd like to see it."

And he produced a *carte de visite*, the very twin of one Cora had but that moment stowed away among her treasures.

"There aint any mistake about *him*, is there?" said the detective, grimly.

"But for what—for what is he—is—is he—arrested?" faltered the poor girl.

"Why, miss, you see he left California too suddenly, with all the spare cash of the proprietor of the Pacific Hotel—thirty thousand dollars—and a matter of five thousand dollars more in jewelry belonging to the boarders of the house."

"But, Mr. Smythe——"

"Smythe! that ain't his name, miss. He's plain Bill Higgins, fancy bar-keeper of the Pacific. I'm very sorry for you, miss. I don't s'pose you had any idea who you were with. Good morning."

She looked at Montgomery Smythe, but he did not raise his eyes nor open his mouth. Plainly the detective had told the truth. She turned to the hotel. Happily the whole affair had escaped notice.

Montgomery Smythe was already on his way to the depot, arm in arm with the stout man, and as they turned a bend in the road, Cora took a last sad farewell look at her prince. The shock made her seriously ill, and when she recovered, the nonsense was pretty thoroughly washed out of her.

Arthur Bayne was not the man to triumph over the fall of any one. On the contrary, he was too generous, and when people began to make remarks about this unfortunate episode in Cora's existence, he married her himself to shut their mouths.

